# Leveraging Twitter and its Unique #HashTag Capability: A Novel Social Media Resource From the European Hernia Society

**DOI:** 10.3389/jaws.2021.10018

**Published:** 2022-01-21

**Authors:** Hakan Gök, Kristian K. Jensen, Maciej Pawlak, Barbora East, Enis Pendar, Shirin Towfigh, Richard Brady, Andrew de Beaux

**Affiliations:** ^1^ European Hernia Society Social Media Advisory Board, Paris, France; ^2^ Hernia Istanbul^®^, Hernia Surgery Center, Istanbul, Turkey; ^3^ Digestive Disease Center, Bispebjerg University Hospital, Copenhagen, Denmark; ^4^ European Hernia Society Website Advisory Board, Paris, France; ^5^ Northern Devon Healthcare NHS Trust, Barnstaple, UK; ^6^ 3rd Department of Surgery, 1st Medical Faculty, Charles University and Motol University Hospital, Prague, Czechia; ^7^ Pendar Management Consulting and Business Development, Istanbul, Turkey; ^8^ Beverly Hills Hernia Center, Beverly Hills, CA, United States; ^9^ Newcastle Centre for Bowel Disease Research Group, Newcastle Upon Tyne Hospitals NHS Foundation Trust, Newcastle Upon Tyne, UK; ^10^ Royal Infirmary of Edinburgh, Edinburgh, UK

**Keywords:** hernia surgery, social media, hernia hashtag, hashtag, European Hernia Society

## Abstract

**Background:** Digital and Social Media (#SoMe) platforms have revolutionized the way information is shared, classified and accessed among medical professionals worldwide. The aim of this study was to review the hashtags used on Twitter by @EuroHerniaS to provide a practical roadmap for easier social media utilization for hernia surgery stakeholders.

**Methods:** The hashtags used in tweets and retweets of the @EuroHerniaS Twitter feed were collated since its foundation in November 2016.

**Results:** The first hashtag used was #HerniaSurgery. Since foundation to July 2021, the @EuroHerniaS Twitter feed has used 90 separate hashtags. The number of new hashtags per year was increasing leading to the development of an online library. The increasing diversity of hernia related hashtags allows for the more detailed posting and searching of hernia related information on the #SoMe platform Twitter.

**Conclusion:** The more detailed use of hashtags on Twitter is to be encouraged. Hernia surgeons can make use of them both when posting and reviewing posts to aid the categorization of posts.

## Introduction

Over the last decade, social media (#SoMe) has played an increasing role in many aspects of our private and professional lives [1]. #SoMe literacy and fluency have become useful skills for medical professionals in order to swiftly follow recent developments as well as communicate, consult and collaborate with colleagues [2]. There are many digital platforms with different approaches, functions, utilities, capabilities and target audiences. The most popular being Facebook, Twitter, Instagram, YouTube and LinkedIn [3]. Twitter enables users to share text, pictures, videos and links at a predetermined character limit (currently 280). This enforced succinctness making Twitter the channel of choice for many types of institutions and leaders, including governments, non-governmental organizations, businesses, politicians and celebrities [4].

One capability of Twitter is the ability to “HashTag” a post. This enables users to easily filter and access previously stacked information. HashTags connect posts of a certain topic under an “umbrella” designated by a name, such as #HerniaSurgery, akin to keywords in digital scientific articles [5]. The Social Media Advisory Board of the European Hernia Society (EHS) and others have been developing effective new capabilities on Twitter, especially with its unique HashTag utility [6]. The utilisation of existing and creation of new HashTags has established an evolving “HashTag library” on the theme of hernia, which enables Twitter users to easily search, reach and share any specific information.

The aim of this paper is to report the various HashTags utilised to date with relevance to #HerniaSurgery. We provide a case demonstration to explain in a step-by-step manner the practical benefit of understanding the HashTag system.

## Methods

EHS is a non-profit organization focusing on abdominal wall surgery since 1979 [7]. We retrospectively reviewed the Twitter feed from @EuroHerniaS to collect all the HashTags used in our tweets and retweets.

## Results

In November 2016, the Twitter account of the European Hernia Society displayed just one HashTag (#HerniaSurgery). Currently, we have 4,000 followers on Twitter from more than 100 different countries worldwide. We generated 4,950 posts from November 2016 to July 2021, corresponding to an average of 2.88 posts per day. We identified 90 separate hernia related HashTags, presented along with a short descriptor of their meaning in Table 1.

**TABLE 1:** List of HashTags.

HashTag name	Description of HashTag
Common	
#HerniaSurgery	The original Hernia HashTag!
#HerniaNews	Hernia news items
#HerniaPrevention	Related to tweets on hernia prevention, ideas, techniques
#HerniaSocietyUncovered	An initiative of the EHS to signpost every National Hernia Society in the world
#HerniaVideo	Tags posts with a “hernia” video
#HerniaRegistry	Posts regard to hernia registiries
#HerniaContentHere	Labeling hashtag for tagging a hernia content
#Herniapedia	A play on Wikipedia for hernias!
#SoMe4Hernia	The #SoMe movement hernia classification HashTag
#MustReadHerniaTrials	An initiative of the EHS to signpost important publications relating to hernia
#HerniaPoll	Posts utilising a poll of Twitter users
#HerniaTrip	Posts involving travel on hernia related activities
#HerniaChat	Posts signposting online chat–abdominal wall closure and hernia prevention two topics covered to date
#HerniaFact	Posts emphasize hernia facts
#Herniamed	Posts regard to German Hernia Registry
#HerniaBasecamp	Online hernia education initiative of Medtronic
#HerniaMesh	Posts related to mesh
About EHS	
#EuroHerniaSnews	News from the European Hernia Society
#EHSnewsletter	The bulletin of the European Hernia Society
#EHSmsc	EHS Member’s Speakers’ Corner–A private Facebook group for EHS members only!
#EHSjClub	Monthly EHS journal club HashTag!
#EHSregistry	Posts signposting and relating to the EHS Registry
#JoAWS	EHS’s own Journal of Abdominal Wall Surgery
Type of Hernia	
#GroinHernia	Posts that are associated with specific hernias–the clue is in the name!
#InguinalHernia	Post relating to inguinal hernia
#FemoralHernia	Post relating to femoral hernia
#ObturatorHernia	Post relating to obturator hernia
#UmbilicalHernia	Post relating to umbilical hernia
#EpigastricHernia	Post relating to epigastric hernia
#SpigelianHernia	Post relating to Spigelian hernia
#IncisionalHernia	Post relating to incisional hernia
#ParastomalHernia	Post relating to parastomal hernia
#LumbarHernia	Post relating to lumbar hernia
#HiatalHernia	Post relating to hiatal hernia
#PerinealHernia	Post relating to perineal hernia
#TrocarSiteHernia	Post relating to trocar site hernia
#SubxiphoidHernia	Post relating to subxiphoid hernia
#SuprapubicHernia	Post relating to suprapubic hernia
#MorgagniHernia	Post relating to Morgagni hernia
#BochdalekHernia	Post relating to Bochdalek hernia
#DiastasisRecti	Post relating to diastasis of the recti
#GiantHernia	Post relating to “giant” hernia
#OpenAbdomen	Post relating to open abdomen
#OAT	Post relating to open abdomen treatment
Hernia techniques	
#TAPP	Posts that are associated with a specific technique–again the clue is in the name!
#TEP	Posts that are associated with total extraperitoneal surgery
#rTAPP	Posts that are associated with robotic transabdominal pre-peritoneal surgery
#eTEP	Posts that are associated with enhanced view total extra-peritoneal surgery
#AWR	Posts that were more generic covering a number of surgical techniques in abdominal wall reconstruction
#CAWR	Posts that were more generic covering a number of surgical techniques in complex abdominal wall reconstruction
#VHR	Posts that were more generic covering a number of surgical techniques in primary ventral hernia repair
#IPOM	Posts that are associated with the IPOM technique
#IPOMplus	Posts that are associated with the IPOMPlus technique
#RoboTAR	Posts that are associated with robotic TAR surgery
#RivesStoppa	Posts that are associated with the retro-muscular technique
#SmallBitesTechnique	A hernia prevention HashTag!
#cvMPO	Critical view of the myopectineal orifice
Hernia event	
#HerniaCongress	Posts signposing hernia conferences
#HerniaEvent	Post signposting any hernia event including conferences, training sessions
#HerniaCourse	Posts signposting hernia training events
#HerniaSymposium	Posts signposting hernia symposiums
#OperationHernia	Posts signposting this charities work and also posts from those taking part in hernia missions
#HerniaHelp	Posts signposting this charities work and also posts from those taking part in hernia missions
#HerniaU	Posts signposting Hernia U events, an initiaitive of BD Bard with EHS approval
#HerniaWebinar	Posts signposing online hernia events
#HerniaLearnLive	Posts signposting virtual hernia events
Guidelines	
#HerniaGuidelines	Posts that reference hernia related guidelines
#IntGroinHerniaGuidelines	Posts that reference the HerniaSurge guidelines including sepcific recommendations
#UmbEpiHerniaGuidelines	Posts that reference the EHS primary ventral hernia guidelines including specific recommendations
#HerniaClassification	Post that reference hernia classifications
Social	
#HerniaFriends	Posts that were more about the hernia surgeon community
#HerniaNerds	Posts that are more about people with a hernia interest
#HerniaGuru	Posts that are more about people with a hernia interest
#HerniaTantra	Posts that are more about people with a hernia interest
#Herniator	Posts that are more about people with a hernia interest
#Herniologist	Posts that are more about people with a hernia interest
#HerniaMaster	Posts that are more about people with a hernia interest
Mesh Problems	
#SurgicalMesh	Posts related to mesh use, by both surgeons and mesh interested groups
#MeshInfection	Posts related to mesh infection
#MeshAwareness	The remaining Hashtags in this section have largely been coined by anti-mesh groups
#PelvicMesh	Post related to anti-mesh use in gynaecological surgery
#SlingTheMesh	Posts related to anti-mesh campaign
#MeshAwarenessDay	Posts related to anti-mesh campaign
#SayNoToMesh	Posts related to anti-mesh campaign
#MeshCampaign	Posts related to anti-mesh campaign
#CPIP	Posts related to chronic post-operative inguinal pain
Slogan	
#ItsNotJUSTaHernia	Posts related to surgical specialisation
#SayNoToPlugs	Posts related to stop plug use by both surgeons and anti-mesh groups
#StopTheBulge	Initiative launched by the Americas Hernia Society (AHS) at their 2019 Annual Meeting
#LetsMakeHerniaGreatAgain	Posts related to promoting quality hernia surgery by those with an interest in hernia surgery

If we give some examples of the most used HashTags from different categories; #HerniaSurgery is the most commonly used HashTag in the “Common” category. It’s original HashTag and dedicated to hernia surgery. Almost all posts are tagged with this HashTag. The activities of the EHS were also tagged and listed in the category “About EHS”. For example, the Hernia Registry, which EHS launched this year, has been tagged as #EHSregistry. Again, EHS’s regular newsletter to members was tagged #EHSnewsletter. EHS has been performing journal club sessions, monthly on the fourth Tuesday at 8 pm CET on Twitter for 1 year and gaining increasing attention. The tag for this event is #EHSjClub.

Hernia types are listed separately under the category “Type of Hernia”. It’s recommended that if the post is related to a specific hernia, should be tagged with its name. The clue is the name itself. Many techniques have been described and applied for hernia repair so far. With the introduction of MIS techniques, acronyms have become more used. We have gathered the tags related to hernia surgery techniques under the tag of “Hernia techniques”. The most famous ones of these are #TAPP and #TEP.

The social media wing of EHS announces the information about all hernia activities worldwide to its followers like a media agency. The HashTags related to this subject have been collected in the “Hernia Event” category like #HerniaCongress if the event is a congress, #HerniaCourse if it’s a hernia course. Hernia guidelines are listed under the category “Hernia Guidelines”. Most of the hernia guidelines published to date have been given specific HashTags. For example; EHS’s primary ventral hernia guidelines have been labeled as #UmbEpiHerniaGuidelines. The HashTags emphasizing the social aspects of the surgeons who dealing with abdominal wall surgery were not forgotten and were gathered under the social title. On a lighter note, #HerniaFriends provides a venue for the equivalent of social “happy hour” online while #HerniaGuru, #HerniNerd and #HerniaMaster is used to honor colleagues. As such, posts relating more to the inter-social relationships of hernia surgeons can be identified using #HerniaFriends and find a place in the online collegial network. Patients suffering from hernia surgery related problems such as chronic pain associated with the meshes are well organized on social media and they also use certain HashTags and slogans frequently. We have collected them in the category of mesh problems.

## Discussion

This study identified a large number of HashTags used by the EHS on its Twitter feed to help characterize Twitter posts related to hernia surgery. At the start of medical professional use of such a #SoMe platform, a simple all-encompassing HashTag such as #HerniaSurgery was sufficient. However, as #SoMe use has grown, both in the number of users and number of tweets and retweets, the need for further HashTags was evident. The EHS has undergone an evolution similar to that of #colorectalsurgery by the European Society of Coloproctology and its eventual need to launch a more specialist HashTag #Colorectalresearch [8] due to volume and need to subspecialize the content for users. We started with relevant information for our membership; conference, symposia and training announcements; abstracts of recent articles; all Hernia Guidelines published to date; clinical studies; current developments in hernia surgery and latest news. Next, we started to provide a platform for collaboration and shared projects by leveraging the capabilities provided by HashTags on Twitter.

One of the more advanced specialties engaging on Twitter utilization was the colorectal surgeons. Wexner et al. showed that #SoMe have a potential positive impact on clinical practice, training, research and patient care in colorectal surgery [3]. Hernia surgeons’ use of #SoMe is increasing, as with other specialties, with a recent study describing that one-third of Danish surgeons actively use #SoMe for professional purposes [9, 10]. Twitter is also widely adopted in the United States, but primarily utilized for promotional content rather than educational [11]. Previous research on hernia surgeons demonstrated that those who use #SoMe in their professional practice are more interested in sharing the speakers’ slides on #SoMe at conferences [12]. Mayol et al. described the potential contributions of #SoMe to surgical research, including widening of international networks and keeping up to date with evolving surgical techniques [13]. Nevertheless, while surgical education is changing rapidly and social media offers tremendous opportunities for teaching, learning, research and networking, guidelines for this purpose would be helpful for trainers and trainees to navigate through this promising area [14].

The use of Twitter to educate and inform has massive potential. The use of HashTags allows a more focused promotion and recall of tweets. Visual content such as images and videos are more effective in attracting attention [15]. Laparoscopic surgery provides ample video content and HashTags for these different surgical techniques are detailed in Table 1. Twitter can respond to events, and the recent COVID-19 pandemic is a good example. Canceled events were replaced with creative “online-response”: which spawned HashTags such as #HerniaWebinar and #HerniaLearnLive [16].

Twitter allows an interface between healthcare workers and patients. Health promotion such as #StopTheBulge, an initiative launched by the Americas Hernia Society, with similar initiatives for hernia charity missions under #OperationHernia and #HerniaHelp [17, 18]. The use of similar HashTags by more than one stakeholder population allows medical and patient groups to overlap, such as #ItsNotJUSTaHernia to highlight that hernia treatment as a whole is not always simple. Some patients do come to harm with hernia surgery, and chronic pain, #CPIP (Chronic Postoperative Inguinal Pain) is a useful HashTag to monitor. There are other more direct advocational hashtags such as #SayNoToPlugs.

### How to Make Practical Research Using HashTags?

**FIGURE 1:**
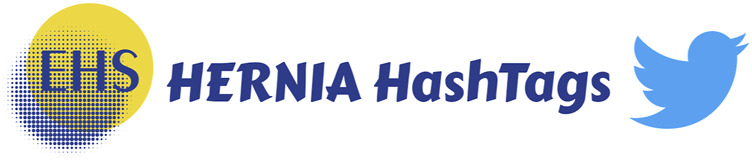
Hashtag image on the website of the EHS.

As an example, let us suppose a surgeon would like to review the latest information related to a specific topic as laparoscopic TEP technique for groin hernia repair. This surgeon could go to the EHS website where there is a Twitter icon with a HashTag icon next to it (Figure 1). When clicked, all available hashtags will be shown in a list in a searchable format. https://www.europeanherniasociety.eu/advantages-becoming-member/hernia-hashtags. The current list provides HashTags that may be relevant #TEP and #GroinHernia. When this is copy pasted to the Twitter search area, all the entries to date will be listed for easy review. Most of those entries contain active links to the original source.

## Conclusion

Surgeons never stop learning and enjoying the intellectual challenge of their colleagues in surgical practice. Social media makes such interactions immediate, international, intimate and easy. A working knowledge of the tenets and processes of Twitter and its HashTag system aids the curation, search and identification of information in a more specific way. The developing language surrounding the use of the known HashTags in hernia surgery assures that an online information evolving repository is created for everyone, helping create a #Herniapedia and building new friendships for those interested in hernia surgery.
